# Analysis and validation of necroptosis-related diagnostic biomarkers associated with immune infiltration in bronchopulmonary dysplasia

**DOI:** 10.3389/fped.2025.1578628

**Published:** 2025-07-15

**Authors:** Haixia Tu, Changjiang Fang, Ping Gan, Yunyun Gu, Nana Peng, Honghua Jiang, Weiwei Hou, Guihua Shu

**Affiliations:** ^1^ Department of Neonatology, Northern Jiangsu People’s Hospital Affiliated to Yangzhou University, Yangzhou, China; ^2^ Department of Pediatrics, The First People’s Hospital of Kunshan, Suzhou, China; ^3^ Department of Neonatology, Yangzhou Maternal and Child Health Care Hospital Affiliated to Yangzhou University, Yangzhou, China

**Keywords:** bronchopulmonary dysplasia, necroptosis, diagnostic biomarkers, immune infiltration, preterm infants

## Abstract

**Background:**

Bronchopulmonary dysplasia (BPD) is the most common serious complication in very preterm infants. This study aims to identify necroptosis-related genes (NRGs) and analyze the relationship between necroptosis-related diagnostic markers and immune infiltration in BPD.

**Methods:**

We obtained the dataset GSE32472 from the GEO database and analyzed the differentially expressed NRGs (DE-NRGs). We identified the biological functions and pathways of DE-NRGs. RF (random forest) and LASSO (least absolute shrinkage and selection operator) algorithms were applied to identify hub genes. We explored the immune landscape of BPD and controls by CIBERSORT. The correlations between hub genes and immune cells were evaluated using Spearman correlation analysis. ELISA was used to verify the diagnostic value of hub genes in patients with BPD in our hospital.

**Results:**

27 DE-NRGs were screened. We found the primary biological functions and pathways of DE-NRGs, including necroptosis, and regulation of inflammatory response. Three hub genes (PELI1, PYGL, and STAT4) were identified and utilized to construct a diagnostic nomogram. The AUC value of the nomogram was greater than 0.7 in the validation dataset GSE188944. CIBERSORT showed that the proportions of 6 different immune cell types in the BPD group were higher or lower than the control group (*P* < 0.05). Correlation analysis showed that three hub genes may correlate with immune cells to varying degrees. Serum ELISA results of PELI1 and PYGL showed no significant difference between BPD and Controls (*P* > 0.05), the expression level of STAT4 was downregulated in BPD samples (*P* < 0.05), and the AUC value of the STAT4 was higher than 0.7.

**Conclusion:**

The necroptosis-related gene STAT4 can be a diagnostic marker of BPD patients. The necroptosis-related gene and immune infiltration may be related to the progression of BPD.

## Introduction

Bronchopulmonary dysplasia (BPD) is a severe chronic respiratory disease in premature neonates. Infants with low birth weight and gestational age are more likely to develop BPD (1). According to a previous study, the main pulmonary pathologies of BPD include immature lung development and acute lung damage (2). Although some breakthroughs have been made in the prevention and treatment of BPD, the current treatments are limited to controlling symptoms and reducing exacerbations. There is still a lack of specific consensus on the diagnosis and management of BPD (3).

Necroptosis is a new mechanism of programmed cell death and combines the relevant features of necrosis and apoptosis (4, 5). The receptor-interacting protein kinases 1 and 3 (RIPK1 and RIPK3) and mixed lineage kinases domain-like pseudo-kinases (MLKL) are important regulators of Necroptosis (6). Previous studies suggested necroptosis-related regulators may lead to cell lysis in acute respiratory distress syndrome and release damage-associated molecular patterns (DAMPs) to initiate the innate immune response (7). Accumulating findings have indicated that necroptosis is engaged in developing cancer (8), respiratory disorders (9), and inflammatory responses (10). The preprocessing of free radical scavengers can inhibit the oxidative stress of acute lung damage and reduce the number of necrotic cells (11). A previous study indicated that immune infiltration also had a crucial influence on the progress of BPD (12). It would be of great significance to explore how immune cell infiltration results in the pathological processes that contribute to lung injury in BPD and to examine the differences in the composition of various immune cells.

However, few studies have focused on the value of necroptosis-related genes (NRGs) in the early diagnosis of BPD. Our study aims to identify necroptosis-related diagnostic markers and investigate the relationship between necroptosis and immune infiltration through bioinformatics. The research process is depicted in Supplementary Figure S1.

## Methods

### Data acquisition and preprocessing

The dataset GSE32472 was obtained from the Gene Expression Omnibus (GEO) database (https://www.ncbi.nlm.nih.gov/geo/). GSE32472 encompassed gene expression data of 299 blood samples (on the 5th, 14th, and 28th days of life). According to the diagnostic criteria, BPD was diagnosed on the 28th days of life (13). This study aims to find early diagnostic markers for BPD. On the 5th and 14th days of life, we collected 197 blood samples for analysis. Excluding the 3 samples with no outcome, we obtained gene expression data of samples (120 BPD and 74 control blood samples) (Supplementary Table S1). Using the quantile normalization function in “limma” R package, the gene expression data of dataset GSE32472 was normalized (14). The outcome was presented as a boxplot. In addition, 159 NRGs were obtained from the KEGG pathway database (https://www.kegg.jp/kegg/pathway.html) (15) 100 NRGs were collected from the Gene Cards database (https://www.genecards.org/) (16) based on a correlation score >1.6. Finally, the two gene profiles were combined to obtain 236 NRGs.

### Screening of differentially expressed NRGs

The gene expression data of 172 NRGs was extracted from the dataset GSE32472, including BPD and control samples. We applied the “limma” R package to identify the differentially expressed necroptosis-related genes (DE-NRGs) between BPD and control groups. The DE-NRGs were screened based on *p* < 0.05 and |logFC| > 0.2. The volcano and box plots were generated with the “ggplot” R package (17).

### Functional enrichment analysis

To investigate the biological functions and pathways of DE-NRGs, we employed Gene Ontology (GO) and Kyoto Encyclopedia of Genes and Genomes (KEGG) pathway functional enrichment analysis with the “clusterProfiler” and “GOplot” R packages (17, 18). GO enrichment analysis included the biological process (BP), cellular component (CC), and molecular function (MF) categories (19). KEGG pathway analysis was used to explore the functions of genes and the high-level genomic information related to those functions (20). The outcomes were presented as a bar graph.

### Protein-protein interaction (PPI) analysis and hub genes identification

To analyze the interactions among the DE-NRGs, we employed the Search Tool for the Retrieval of Interacting Genes (STRING) online database (https://string-db.org/) (21). The DE-NRGs were used to construct a PPI network, based on a total score >0.4. Then, two machine-learning algorithms were used to identify hub genes. We implemented the LASSO (least absolute shrinkage and selection operator) (22) algorithm to reduce dimensionality and screen the most relevant variables using the “glmnet” R package. Another machine-learning algorithm random forests (RF) (23) was conducted with the “randomForest” R package, and the top five genes were screened for subsequent analysis. According to the two algorithms, the optimal BPD diagnostic biomarkers were determined. The correlations of the hub genes were analyzed using the Spearman correlation in the “corrplot” package (24).

### Diagnostic value of hub genes on the 5th, and 14th days of life

To investigate the expression patterns of hub genes on the 5th (time A), and 14th (time B) days of life in the dataset GSE32472. At time A, we obtained 62 BPD samples and 35 control samples (dataset A). At time B, we obtained 58 BPD and 39 control samples (dataset B). The outcomes were presented as a box plot. Additionally, ROC (receiver operating characteristic) curves were used to assess the diagnostic value of hub genes in BPD, and ROC curve analysis was conducted with the “pROC” R package.

### Construction and validation of the nomogram model

According to the screened marker genes, a nomogram model was established through the “rms” R package. We then generated a calibration curve to assess the coherence between our realistic outcomes and predicted values. Additionally, we conducted a Decision Curve Analysis (DCA) to evaluate whether the decisions made by our model were supportive of patient care. As an external validation dataset, GSE188944 included 17 control samples and 6 BPD umbilical cord tissue blood from preterm infants. The ROC curve analysis was used to evaluate the diagnostic capacity of the nomogram model in the validation dataset GSE188944. The area under the curve (AUC) combines sensitivity and specificity to validate the predictive efficacy of the model (24). The model can have a good predictive value if the AUC value is higher than 0.7.

### Analysis of immune cell infiltration

CIBERSORT (cell-type identification by estimating relative subsets of RNA transcripts) is a way to calculate the proportions of various cell types in the immune microenvironment according to gene expression data (25). In this study, we utilized the CIBERSORT algorithm to estimate the relative proportion of 22 types of immune cells in blood samples in GSE32472. In addition, the “corrplot” R package was used to analyze the correlations among different immune cells. The correlations between hub genes and immune cells were evaluated using Spearman correlation analysis.

### Experimental verification

To further validate the diagnostic value of hub genes, we collected 30 BPD and 21 control blood samples in our hospital (from April to September 2024) (Supplementary Table S2). This study was approved by the Ethics Committee of Northern Jiangsu People's Hospital Affiliated to Yangzhou University (2024ky015). The human enzyme-linked immunosorbent assay (ELISA) kits (PELI1, PYGL, and STAT4) were purchased from Lianmai Bioengineering Corp. (Shanghai, China). Expression levels of PELI1, PYGL, and STAT4 in serum were determined using ELISA. The experimental procedures were conducted according to the manufacturer's instructions.

### Statistical analysis

All statistical analyses were performed using R software (version 4.3.3) and SPSS 28.0 (SPSS, Inc., Chicago, IL, USA). Student's *t*-test was used to compare the mean values between the BPD and control groups. The receiver operating characteristic (ROC) curve was used to evaluate the diagnostic accuracy of hub genes and combined the area under the ROC curves (AUROC), 95% CI, specificity, and sensitivity. Spearman correlation coefficient was used to analyze the associations between hub genes and immune cells. *P* < 0.05 was considered statistically significant.

## Results

### Recognition of DE-NRGs

The gene expression data were normalized using the “limma” R package (Supplementary Figures S2A, S2B). According to dataset GSE32472 and 236 NRGs, we obtained 172 overlapping genes (Supplementary Figure S3A). The volcano plot depicted the differential expression of 27 DE-NRGs (Supplementary Figure S3B). 13 genes were upregulated in the BPD group, whereas 14 genes were downregulated (Supplementary Figures S3C, S3D).

### Analysis of enrichment annotations

We conducted GO and KEGG enrichment analyses to gain a deeper understanding of the functional annotations of the 27 DE-NRGs. GO enrichment analysis showed that the necroptotic process, programmed necrotic cell death, regulation of programmed necrotic cell death, regulation of inflammatory response, and response to the virus were significantly enriched in biological functions (Supplementary Figure S4A). In the KEGG enrichment analysis, the enriched pathways were mainly necroptosis, lipid and atherosclerosis, tuberculosis, etc (Supplementary Figure S4B).

### PPI network construction and hub gene identification

**Figure 1:**
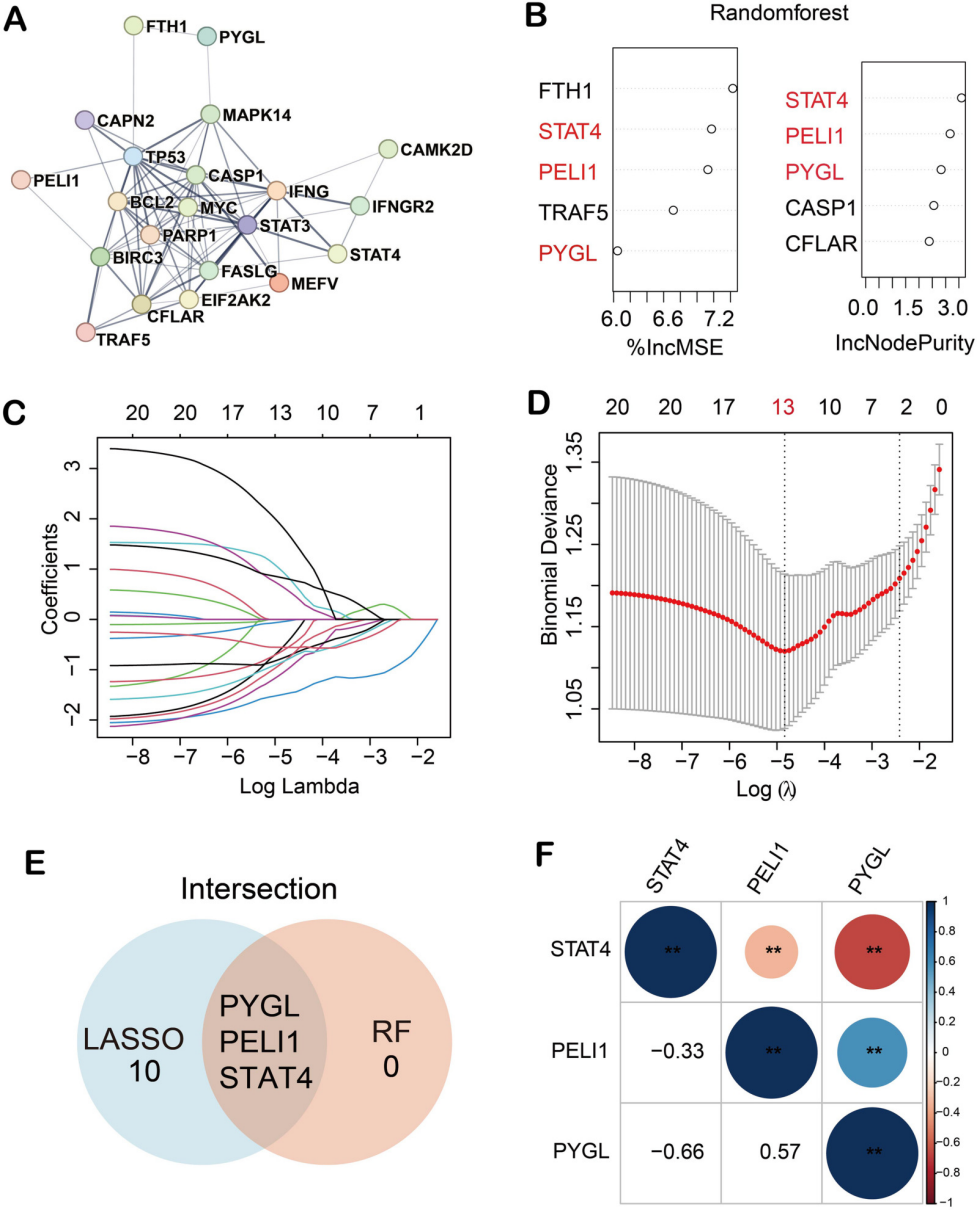
Three hub genes were recognized as biomarkers of BPD. **(A)** 21 DE-NRGs were involved in forming a protein molecule network; **(B)** 3 key DE-NRGs were obtained by RF algorithm; **(C,D)** 13 diagnostic markers were screened by LASSO algorithm; **(E)** 3 biomarkers were obtained by RF and LASSO algorithms; **(F)** Correlations among the three marker genes. (**p < 0.01).

A PPI network was established using the STRING online database (Figure 1A), and 21 DE-NRGs were involved in forming a protein molecule network. To further identify the robust BPD biomarkers from the 21 DE-NRGs, we then conducted two machine learning algorithms to screen the key DE-NRGs of BPD. Random forest (RF) containing two algorithms, top 5 genes were selected by two algorithms (%IncMSE and IncNodePurity), and the two algorithms were combined to obtain 3 DE-NRGs (Figure 1B). Then, 13 marker genes (PYGL, MAPK14, CFLAR, STAT3, IFNGR2, PELI1, FTH1, MEFV, EIF2AK2, STAT4, MYC, TP53, BCL2, CAMK2D, FASLG, IFNG, CAPN2, BIRC3, PARP1, CASP1, TRAF5) were selected by the LASSO algorithm with 10-fold cross-validation (Figures 1C,D). Finally, we obtained 3 marker genes through LASSO and RF algorithms (Figure 1E). The heatmap demonstrated the correlations among the three marker genes (PELI1, PYGL, and STAT4) (Figure 1F).

### Diagnostic value of hub genes on the 5th, and 14th days of life

In the dataset GSE32472, we investigated the differential expression of hub genes on the 5th and 14th days of life. Based on dataset A and dataset B, the expression levels of PELI1 and PYGL were upregulated in BPD samples compared to control samples; the expression levels of STAT4 were downregulated in BPD samples (Supplementary Figures S5A, S5B). The differential expression of the three diagnostic biomarkers between BPD and control samples was statistically significant (*p* < 0.05). Moreover, ROC curves were used to assess the diagnostic capacity of the three hub genes in diagnosing BPD (Supplementary Figure S5C, S5D). On the 5th day, the AUC values of the three hub genes were as follows: PELI1, AUC = 0.748; PYGL, AUC = 0.772; STAT4, AUC = 0.787. On the 14th day, the AUC values of the three hub genes were as follows: PELI1, AUC = 0.645; PYGL, AUC = 0.680; STAT4, AUC = 0.738. The AUC values of STAT4 were higher than 0.7 on the 5th and 14th days of life.

### Establishment and validation of a nomogram

**Figure 2:**
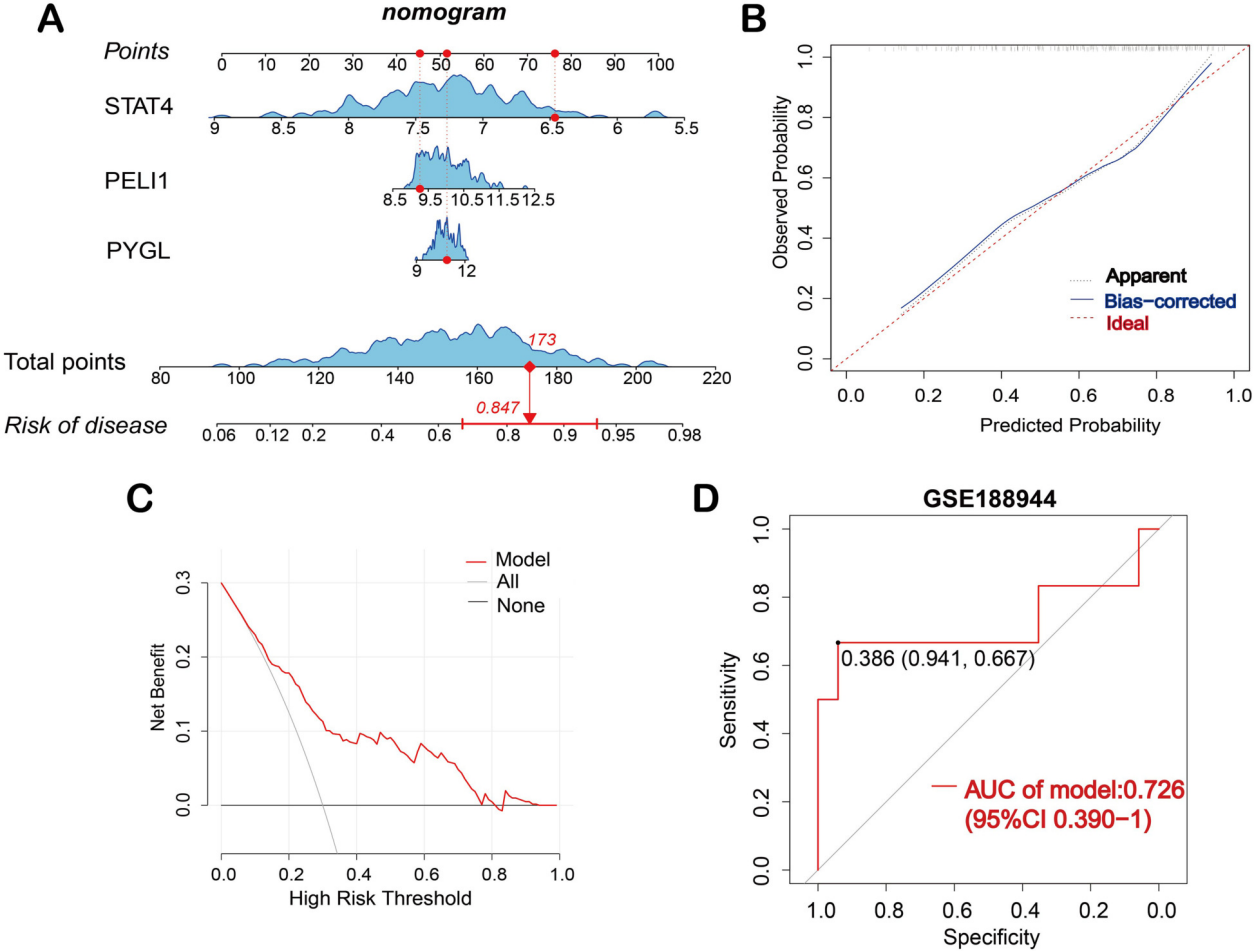
Construction of the nomogram model. **(A)** Nomogram is used to predict the occurrence of BPD; **(B)** Calibration curve demonstrates the nomogram's predictive capability. **(C)** DCA curve shows the nomogram may be used to make decisions that support BPD patients. **(D)** ROC curve for the diagnostic model of BPD in the validation dataset GSE188944.

Based on the three hub genes, a nomogram model was developed to predict the risk of BPD (Figure 2A). The nomogram calibration curve depicted that the prediction group was consistent with the observation group (Figure 2B). The DCA curve showed that decisions based on the model were favorable to the BPD patients (Figure 2C). Subsequently, based on the validation dataset GSE188944, the ROC curve was used to evaluate the diagnostic capacity of the model. The area under the ROC curve (AUROC) of the model reached 0.726 (95% CI 0.390–1), with sensitivity and speciﬁcity of 0.667 and 0.941, respectively (Figure 2D).

### Analysis of immune cell infiltration

In dataset GSE32472, CIBERSORT was applied to calculate the proportions of various cell types in the immune microenvironment of BPD. As shown in Supplementary Figure S6A, the proportions of M0 macrophages and neutrophils were elevated in the BPD samples compared to the control samples (*p* < 0.001), whereas, the proportions of CD8T cells, CD4 naive T cells, CD4 resting memory T cells, and M2 macrophages were elevated in the control samples compared to the BPD samples (*p* < 0.001).

### Correlation analysis

The correlations among different infiltrating immune cells are shown in Supplementary Figure S6B. Neutrophils were positively correlated with activated mast cells but negatively correlated with CD8T cells and CD4 resting memory T cells. CD8T cells were positively correlated with CD4 naive T cells, activated NK cells were positively correlated with activated dendritic cells. CD4 resting memory T cells were negatively correlated with M0 macrophages. The correlations between three hub genes and infiltrating immune cells are shown in Supplementary Figure S7. PELI1 was positively correlated with M0 macrophages and neutrophils but inversely correlated with memory B cells and CD8T cells. PYGL was positively correlated with M0 macrophages and neutrophils but inversely correlated with CD8T cells, CD4 resting memory T cells, and CD4 naive T cells. STAT4 was positively correlated with CD4 naive T cells, CD4 resting memory T cells, and CD8T cells but inversely correlated with M0 macrophages and neutrophils.

### Experimental validation

**Figure 3:**
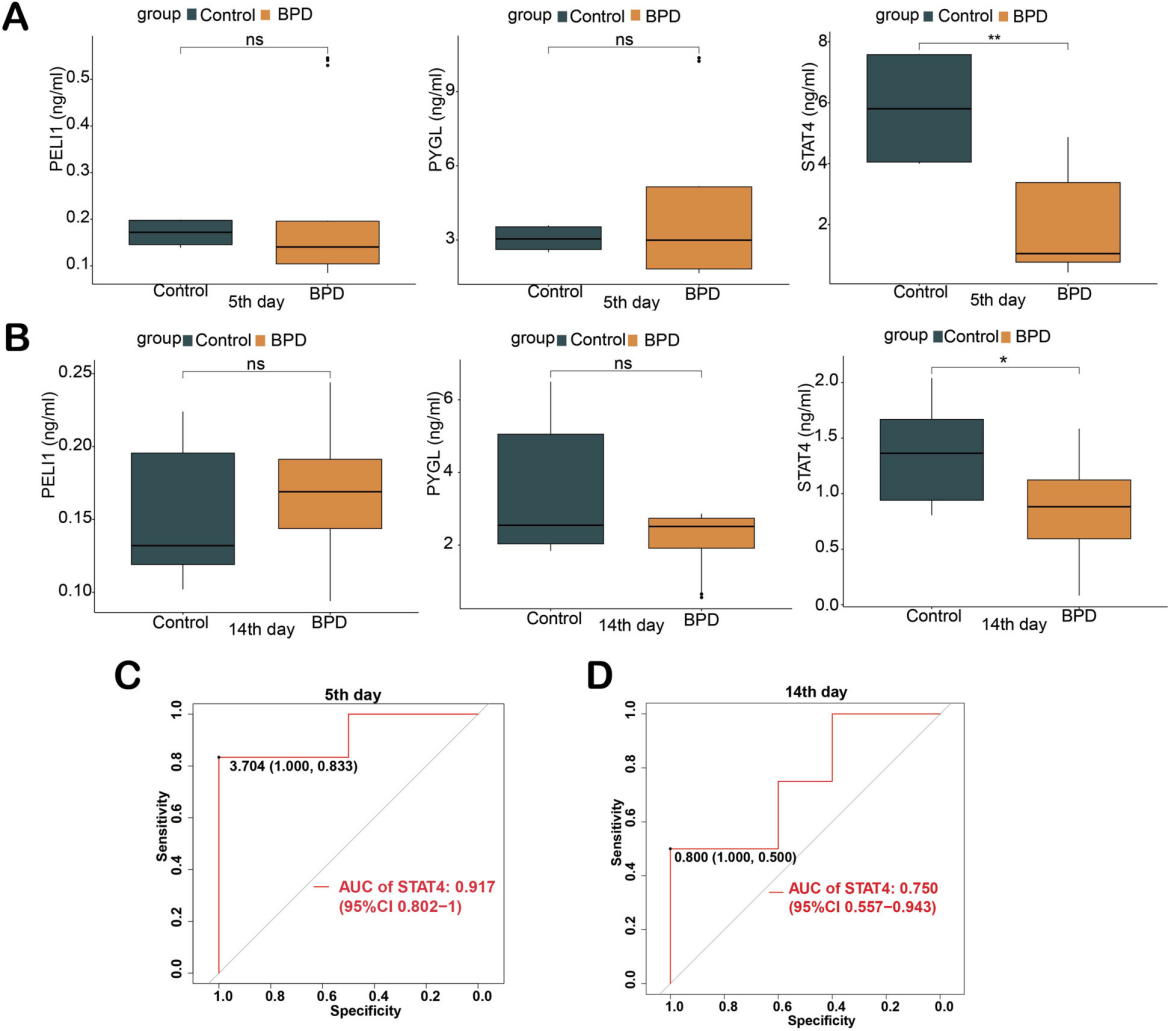
Validation of three hub genes in diagnosing BPD by ELISA. **(A)** Concentration of three hub proteins in serum on the 5th day; **(B)** Concentration of three hub proteins in serum on the 14th day; **(C,D)** ROC curves of STAT4 in diagnosing BPD on the 5th and 14th days of life. (ns *p* > 0.05, *p < 0.05, **p < 0.01).

The expression levels of the three proteins in serum between BPD and control were validated by ELISA. On the 5th day, the expression level of STAT4 was significantly lower in patients with BPD than in controls (median 1.050 vs. 5.802 ng/ml, *p* < 0.01). On the 14th day, the expression level of STAT4 was significantly lower in patients with BPD than in controls (median 0.882 vs. 1.364 ng/ml, *p* < 0.05), whereas PELI1 (*p* > 0.05) and PYGL (*p* > 0.05) were not significantly different at the two different time points (Figures 3A,B). At the two different time points, ROC curves were used to assess the diagnostic value of STAT4 in BPD. On the 5th day, the area under the ROC curve (AUROC) of STAT4 was 0.917 (95% CI 0.802–1), with sensitivity and speciﬁcity of 0.833 and 1.000, respectively. On the 14th day, the AUROC of STAT4 was 0.750 (95% CI 0.557–0.943), with sensitivity and speciﬁcity of 0.500 and 1.000, respectively. (Figures 3C,D).

## Discussion

BPD is one of the most severe complications of newborns, it can affect the quality of life in preterm infants seriously (26). Most patients may even develop emphysema, pulmonary hypertension, and other long-term pulmonary complications (27). Previous studies have indicated that necroptosis is involved in the occurrence and development of a variety of respiratory diseases, such as acute lung injury (ALI), lung infections, chronic obstructive pulmonary disease (COPD), and pulmonary arterial hypertension (PAH) (28). Hyperoxic-induced acute lung injury is one of the most significant factors of BPD, and necroptosis has a crucial effect on this process (29). This study aims to identify effective necroptosis diagnostic biomarkers for BPD patients, which may give insight into the early diagnosis and intervention of BPD.

In our research, the gene expression data of BPD and control groups in GSE32472 were utilized for differential analysis to obtain 27 DE-NRGs (13 up-regulated and 14 down-regulated), which suggested that NRGs may be engaged in the progress of BPD. In addition, GO and KEGG enrichment analyses revealed that 27 DE-NRGs were significantly correlated with necroptotic processes, programmed necrotic cell death, and inflammatory response. We utilized two machine-learning algorithms and identified three hub genes (PELI1, PYGL, and STAT4). Studies have shown that Pellino E3 ubiquitin-protein ligase 1 (PELI1) can specifically regulate the aging marker p21. In the D-galactose aging mouse model, inhibiting the expression of PELI1 can inhibit the aging of pulmonary tissue cells mediated by p21 and oxidative stress, inflammatory response (30). According to a previous study, in the chronic obstructive pulmonary disease (COPD) mouse model, inhibiting the expression of PELI1 can reduce senescence-associated secretion phenotypes (SASPs) regulated by p21. Thus, it can inhibit the inflammatory response in COPD (31). Some scholars found that the expression level of glycogen phosphorylase L (PYGL) in lung cancer tissue is significantly higher than that in normal lung tissue, and the expression level of PYGL is positively correlated with the poor prognosis of lung cancer patients. Low expression of PYGL can significantly inhibit the proliferation and migration of lung cancer cells (32). Other studies have shown that high expression of PYGL is associated with poor prognosis in patients with lung adenocarcinoma (33). Relevant studies have shown that in the BPD lung tissue of newborn mice, with the increase of DNA methylation, the expression level of signal transducer and activators of transcription factor 4 (STAT4) is significantly lower than that in the control group, indicates that STAT4 may be regulated by DNA methylation in the occurrence and development of BPD (34). In this study, in the dataset GSE32472, the expression of PELI1 and PYGL was upregulated in the BPD group, while STAT4 was down-regulated in the BPD group, suggesting that necroptosis-related genes PELI1 and PYGL may act as pro-inflammatory factors, while STAT4 is an anti-inflammatory factor, the three hub genes may participate in the inflammatory response of BPD through the necroptosis mechanism.

Based on the three marker genes, a diagnostic nomogram model was established to predict the risk of BPD. In the validation dataset GSE188944, the ROC curve showed that the AUC value of the model was greater than 0.7, and the model had a good diagnostic capacity in BPD. In addition, ELISA was used to verify the diagnostic value of the three hub genes in BPD patients. On the 5th and 14th day, serum ELISA results of PELI1 and PYGL showed no significant difference between BPD and control samples. The expression level of STAT4 was downregulated in BPD samples. The AUC values of STAT4 were higher than 0.7 on the 5th and 14th days of life. The ROC curves showed that STAT4 had a good diagnostic capacity in BPD. However, due to the limited number of samples, the AUC value of the hub genes should be validated with larger sample sizes in future studies.

Emerging evidence suggests that immune response may be involved in chronic lung illness in preterm infants (35, 36). A previous study showed that T lymphocytes were crucial in chronic pulmonary disease in preterm infants (37). A high neutrophil-to-lymphocyte rate was an early potential marker of BPD and can act as a predictive factor of intrauterine infection in preterm infants (38). In this study, we calculated the abundance of immune cells based on the microarray profiles of control and BPD blood samples by CIBERSORT. In dataset GSE32472, six types of immune cells were significantly different between BPD and control groups. This finding suggested that immune cell infiltration may be related to the progression of BPD. In addition, Spearman correlation analysis showed that three hub genes may correlate with immune cells to varying degrees. This finding indicated that necroptosis-related genes may be involved in the progression of BPD through immune mechanism.

Our research has some limitations. First, there was a lack of signal pathway-related mechanisms for further verification. Second, there were not enough *in vivo* and *in vitro* experiments to verify the level of expression of the hub genes, which reduced the reliability of our research. Besides, the sample size was relatively small, and large cohorts are needed to confirm our results.

## Conclusion

The necroptosis-related gene STAT4 can be a candidate diagnostic marker for BPD patients. The necroptosis-related gene and immune infiltration may be related to the progression of BPD. However, future studies of necroptosis in patients with BPD need to be further meticulously explored.

## Data Availability

The datasets presented in this study can be found in online repositories. The names of the repository/repositories and accession number(s) can be found in the article/Supplementary Material.

## Supplementary material

Supplementary Figure 1 A flowchart of the investigation process.

Supplementary Figure 2 The gene expression data of dataset GSE3247 was normalized. **(A,B)** The distribution of the sample values is centered around the median and basically lies on the same straight line, it indicates that the data has been standardized and is comparable.

Supplementary Figure 3 Identification of differentially expressed NRGs. **(A)** The Venn diagram shows the intersection of genes in the GSE32472 and NRGs; **(B)** The volcano plot depicts the differential expression between the BPD and control groups; **(C)** Box plot of 13 upregulated DE-NRGs; **D** Box plot of 14 downregulated DE-NRGs. (**p* < 0.05, ***p* < 0.01, ****p* < 0.001).

Supplementary Figure 4 Functional enrichment analysis of DE-NRGs. **(A)** GO enrichment analysis includes the biological process (BP), cellular component (CC), and molecular function (MF) categories. **(B)** KEGG enrichment analysis shows the top 10 significant signaling pathways.

Supplementary Figure 5 On the 5th and 14th days of life, the diagnostic value of three hub genes in dataset GSE32472. **(A,B)** The expression levels of the three diagnostic biomarkers in dataset A and dataset B; **(C,D)** ROC curves were generated for the three hub genes on the 5th and 14th days of life (**p* < 0.05, ***p* < 0.01, ****p* < 0.001).

Supplementary Figure 6 Immune infiltration analysis. (**A**) The Box plot shows the proportions of immune cell types between BPD and control samples in dataset GSE32472; (**B**) Heatmap of the correlation of different immune cells. (ns *p* >0.05, **p* < 0.05, ***p* < 0.01, ****p* < 0.001).

Supplementary Figure 7 The correlation between immune cells and three hub genes (**A**-**C**). The size of the dot indicates the strength of the association between the gene and the immune cell; a larger dot indicates a stronger correlation. The dot's color represents the *p*-value; the more yellow the color is, the smaller the *p*-value.
