# Supplementary figures and images for: Analysis and validation of necroptosis-related diagnostic biomarkers associated with immune infiltration in bronchopulmonary dysplasia
Source: Front Pediatr. 2025 Jul 15;13:1578628. doi: 10.3389/fped.2025.1578628 (PMC12303952; doi:10.3389/fped.2025.1578628)

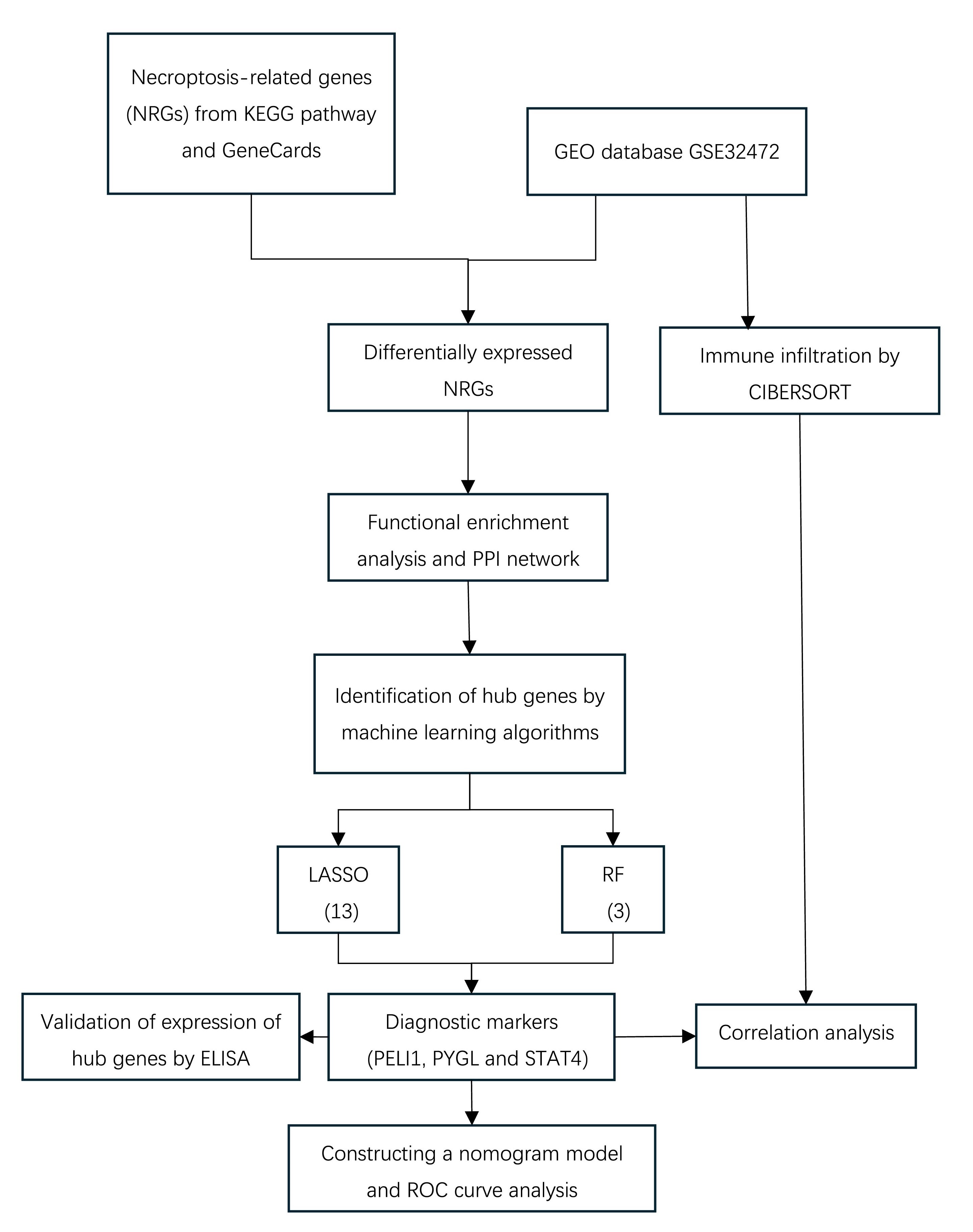

Supplement: Supplementary Figure 1 — A flowchart of the investigation process. [file Image1.jpg]

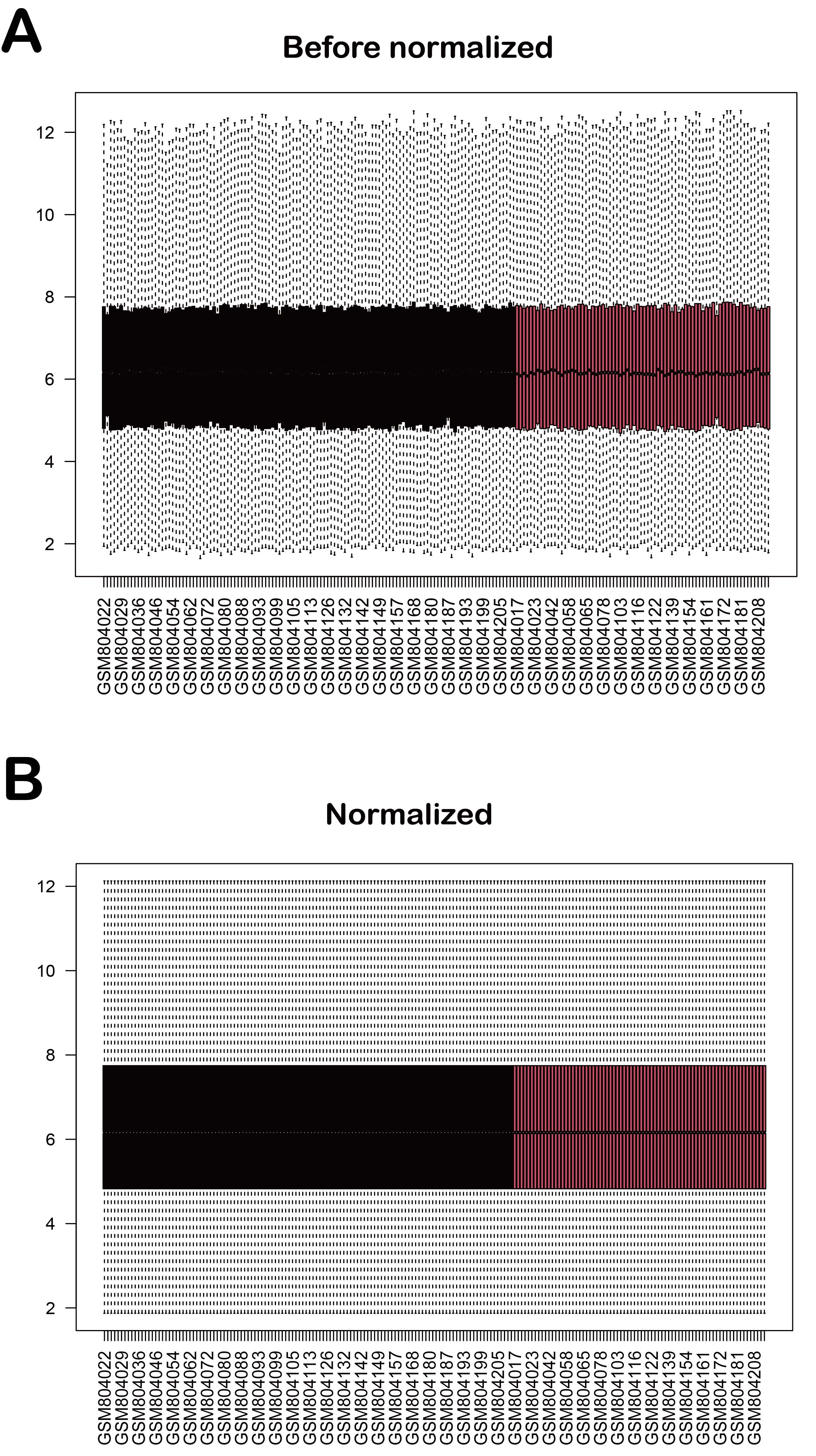

Supplement: Supplementary Figure 2 — The gene expression data of dataset GSE3247 was normalized. (A,B) The distribution of the sample values is centered around the median and basically lies on the same straight line, it indicates that the data has been standardized and is comparable. [file Image2.jpg]

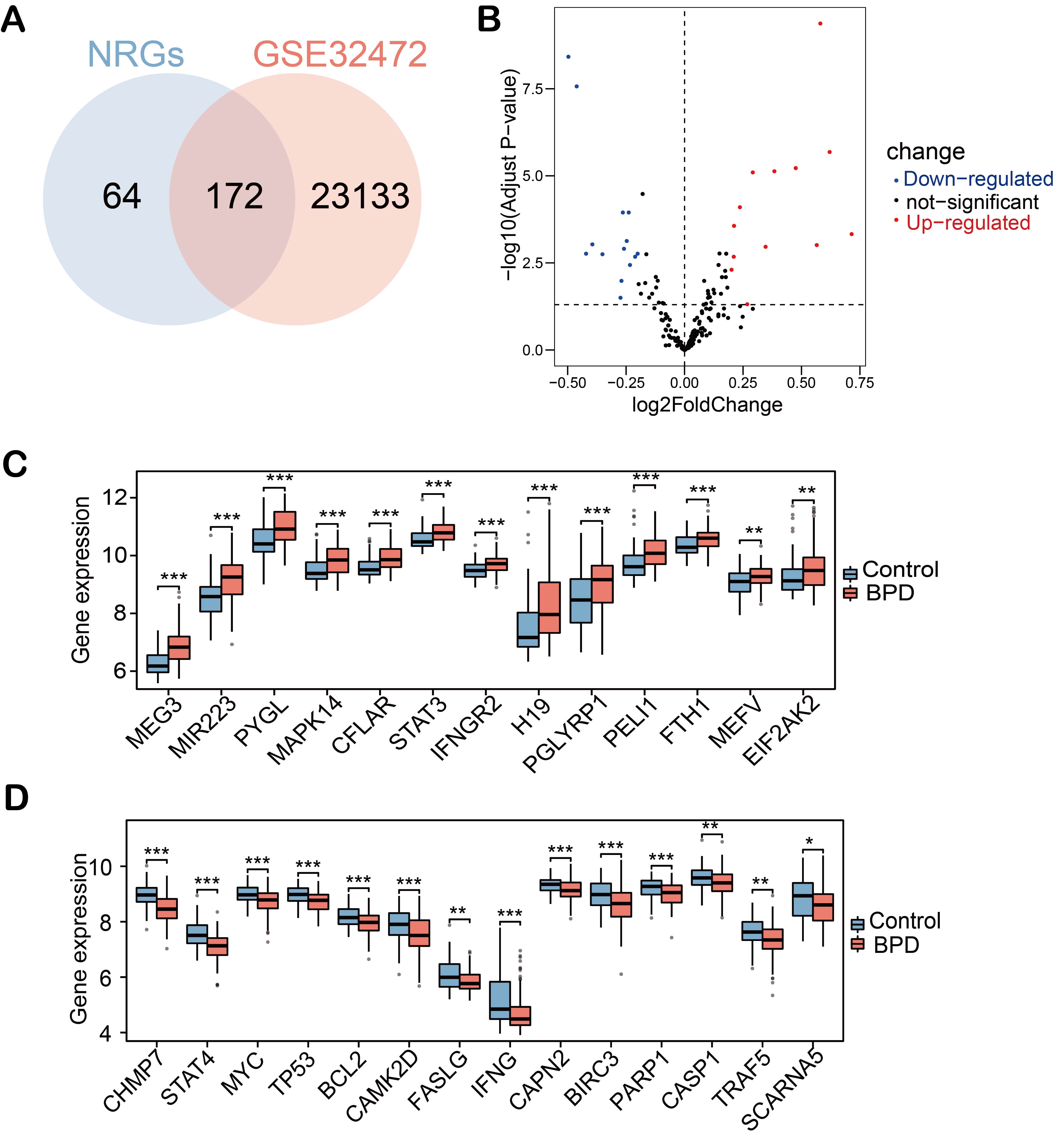

Supplement: Supplementary Figure 3 — Identification of differentially expressed NRGs. (A) The Venn diagram shows the intersection of genes in the GSE32472 and NRGs; (B) The volcano plot depicts the differential expression between the BPD and control groups; (C) Box plot of 13 upregulated DE-NRGs; D Box plot of 14 downregulated DE-NRGs. (*p < 0.05, **p < 0.01, ***p < 0.001). [file Image3.jpg]

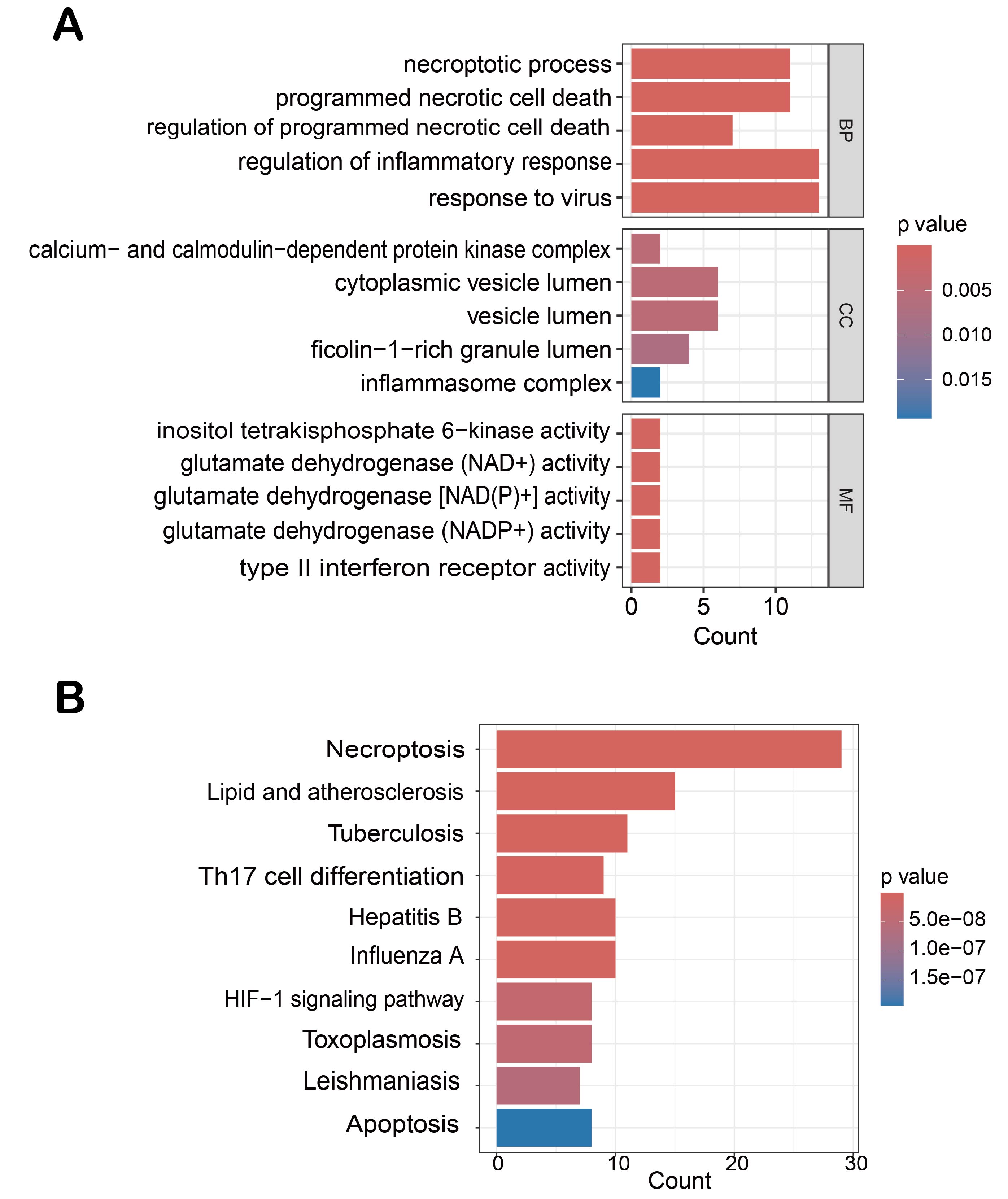

Supplement: Supplementary Figure 4 — Functional enrichment analysis of DE-NRGs. (A) GO enrichment analysis includes the biological process (BP), cellular component (CC), and molecular function (MF) categories. (B) KEGG enrichment analysis shows the top 10 significant signaling pathways. [file Image4.jpg]

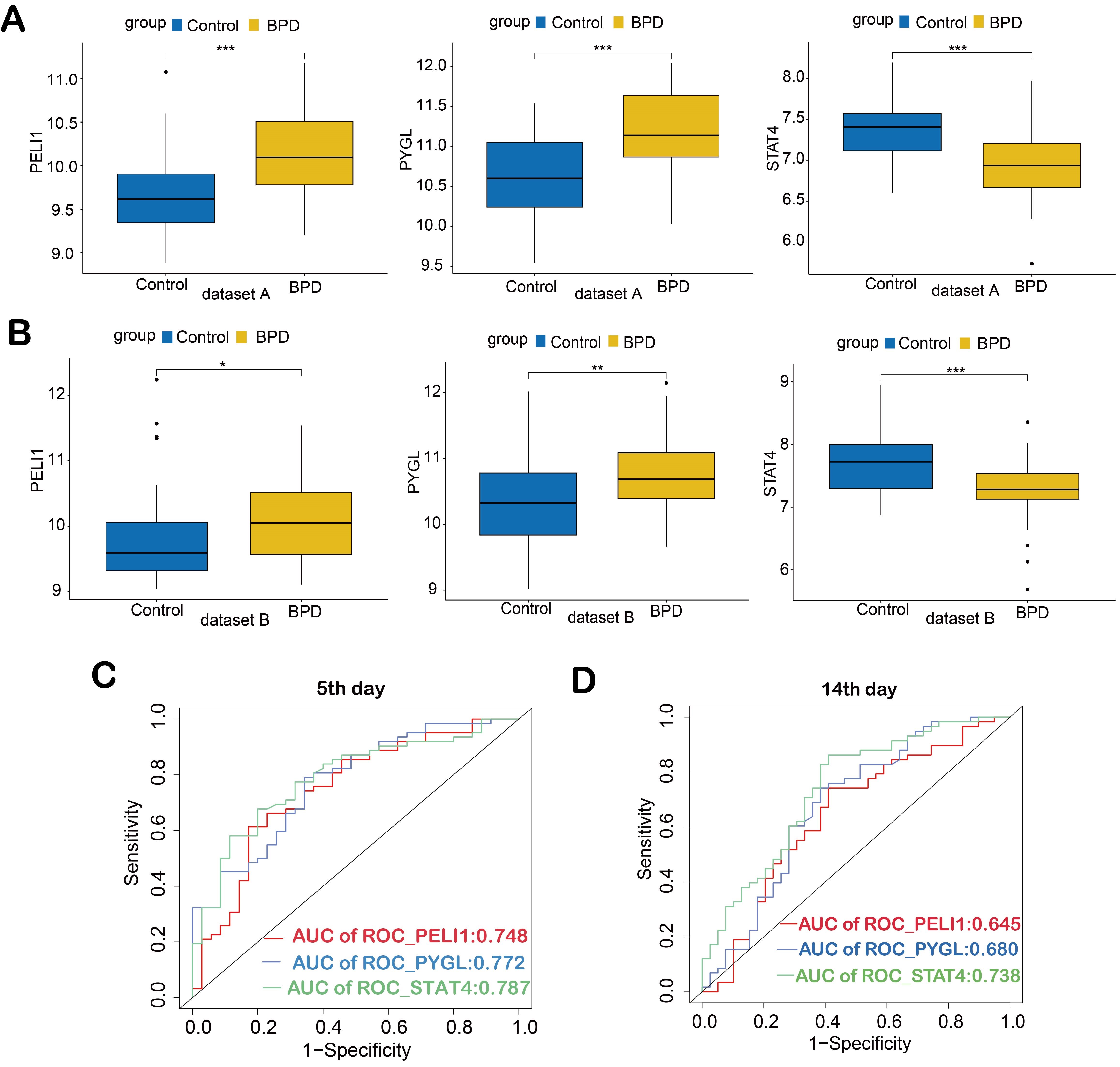

Supplement: Supplementary Figure 5 — On the 5th and 14th days of life, the diagnostic value of three hub genes in dataset GSE32472. (A,B) The expression levels of the three diagnostic biomarkers in dataset A and dataset B; (C,D) ROC curves were generated for the three hub genes on the 5th and 14th days of life (*p < 0.05, **p < 0.01, ***p < 0.001). [file Image5.jpg]

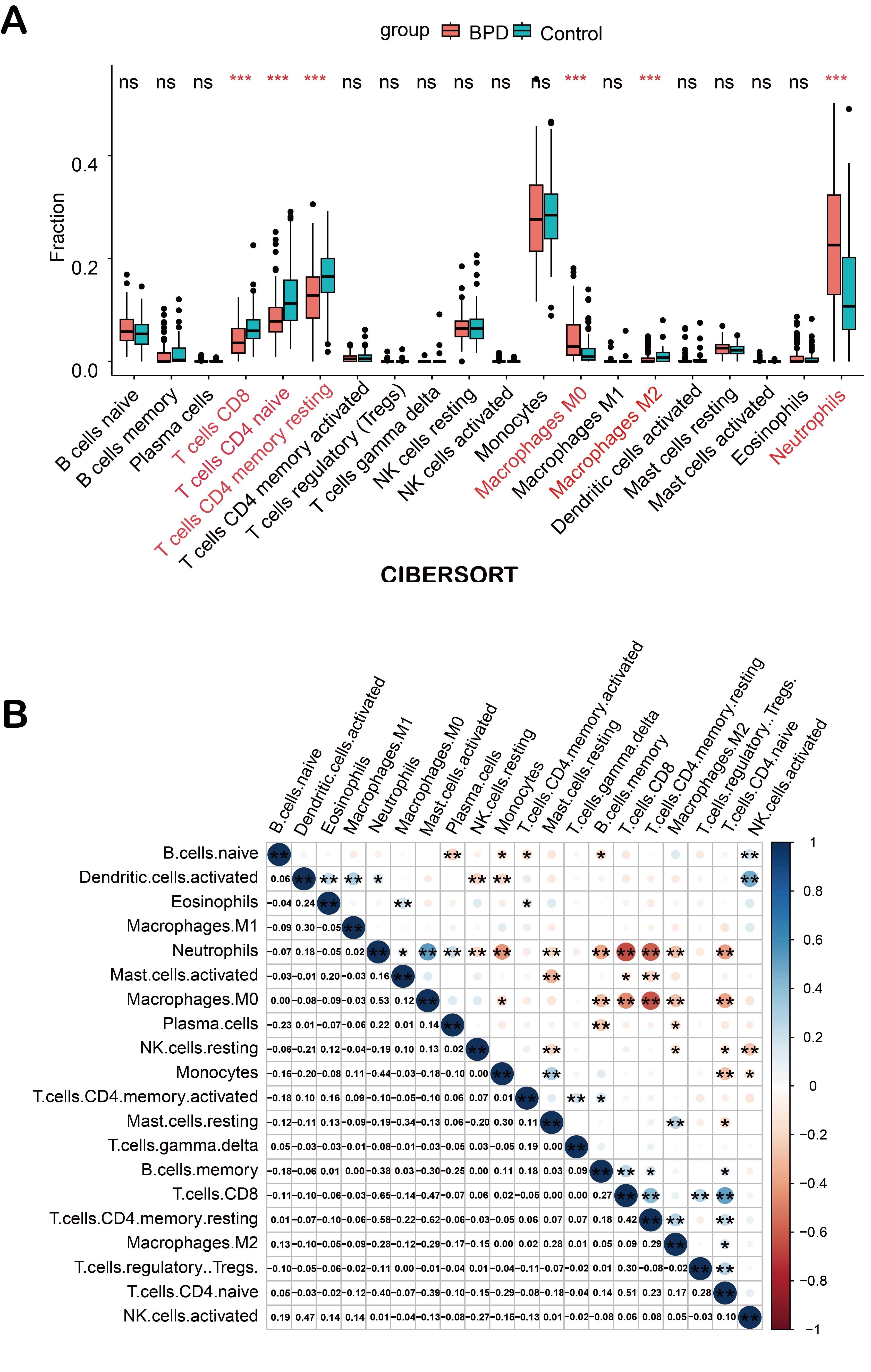

Supplement: Supplementary Figure 6 — Immune infiltration analysis. (A) The Box plot shows the proportions of immune cell types between BPD and control samples in dataset GSE32472; (B) Heatmap of the correlation of different immune cells. (ns p >0.05, *p < 0.05, **p < 0.01, ***p < 0.001). [file Image6.jpg]

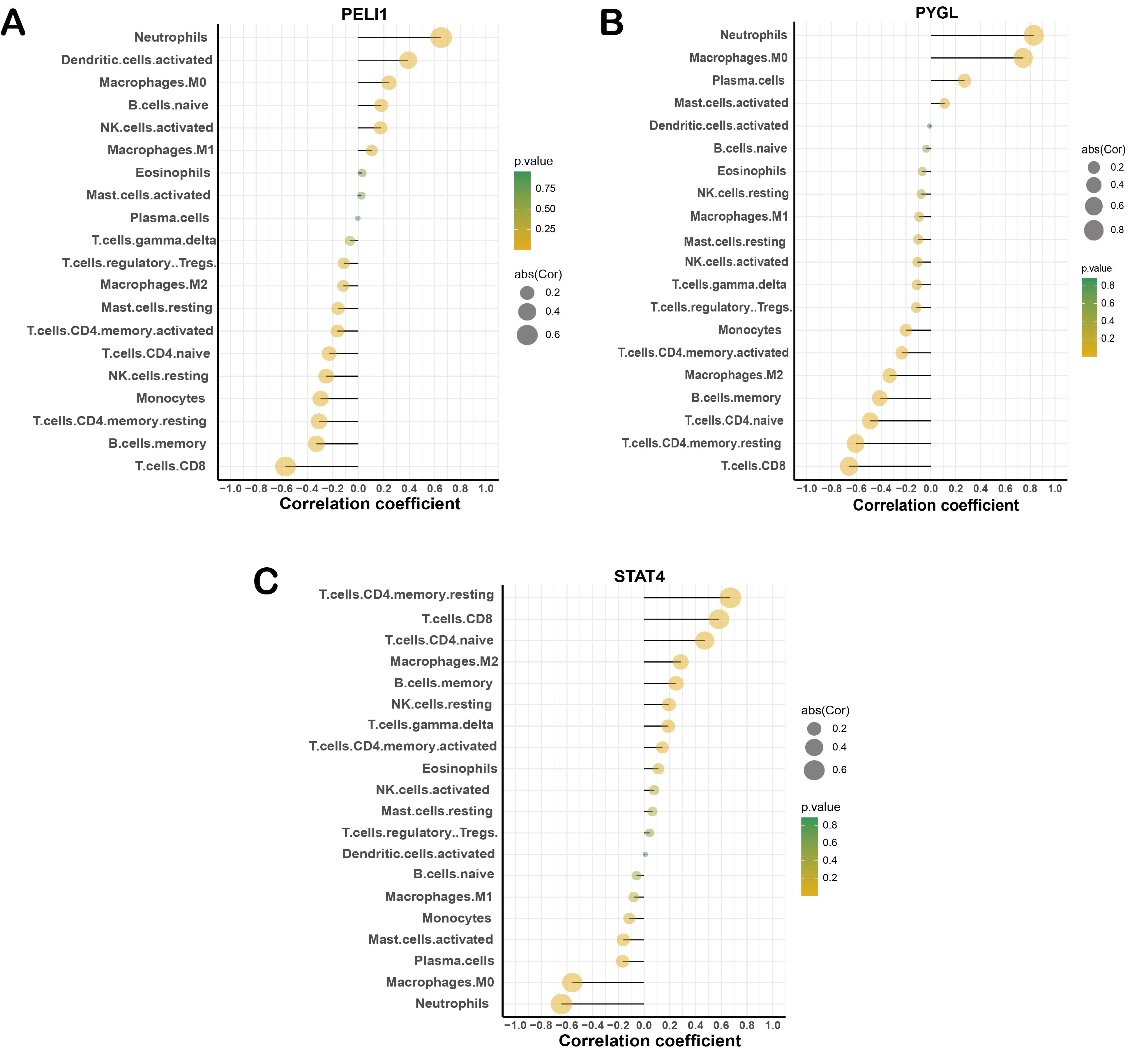

Supplement: Supplementary Figure 7 — The correlation between immune cells and three hub genes (A-C). The size of the dot indicates the strength of the association between the gene and the immune cell; a larger dot indicates a stronger correlation. The dot's color represents the p-value; the more yellow the color is, the smaller the p-value. [file Image7.jpg]
