# Supplementary material for: Analysis and validation of necroptosis-related diagnostic biomarkers associated with immune infiltration in bronchopulmonary dysplasia
Source: Front Pediatr. 2025 Jul 15;13:1578628. doi: 10.3389/fped.2025.1578628 (PMC12303952; doi:10.3389/fped.2025.1578628)
Supplement: Supplementary file 8 [file Table1.docx]

Supplementary Table 1. The characteristics of dataset GSE32472 on the 5th and 14th days of life.

| **GSE32472** | **species** | **platform** | **sample** | **BPD** | **control** | | **application** |
| --- | --- | --- | --- | --- | --- | --- | --- |
| 5d | Homo sapiens | GPL6244 | blood | 62 | 35 | analysis | |
| 14d | Homo sapiens | GPL6244 | blood | 58 | 39 | analysis | |

Supplementary Table 2. The characteristics of two groups of patients on the 5th and 14th days of life.

| **time** | **species** | **age** | **sample** | **BPD** | **Control** | **application** |
| --- | --- | --- | --- | --- | --- | --- |
| 5d | Homo sapiens | neonatal | blood | 18 | 6 | validation |
| 14d | Homo sapiens | neonatal | blood | 12 | 15 | validation |
